# Balancing brain metabolic states during sickness and recovery sleep

**DOI:** 10.1111/ejn.16588

**Published:** 2024-11-14

**Authors:** Sara B. Noya, Arjun Sengupta, Zhifeng Yue, Aalim Weljie, Amita Sehgal

**Affiliations:** ^1^ Howard Hughes Medical Institute University of Pennsylvania Philadelphia Pennsylvania USA; ^2^ Chronobiology and Sleep Institute, Perelman School of Medicine University of Pennsylvania Philadelphia Pennsylvania USA; ^3^ Department of Systems Pharmacology and Translational Therapeutics University of Pennsylvania Philadelphia Pennsylvania USA; ^4^ Institute for Translational Medicine and Therapeutics University of Pennsylvania Philadelphia Pennsylvania USA

**Keywords:** brain, LPS, metabolomics, sleep deprivation

## Abstract

Sickness sleep and rebound following sleep deprivation share humoral signals including the rise of cytokines, in particular interleukins. Nevertheless, they represent unique physiological states with unique brain firing patterns and involvement of specific circuitry. Here, we performed untargeted metabolomics of mouse cortex and hippocampus to uncover changes with sickness and rebound sleep as compared with normal daily sleep. We found that the three settings are biochemically unique with larger differences in the cortex than in the hippocampus. Both sickness and rebound sleep shared an increase in tryptophan. Surprisingly, these two sleep conditions showed opposite modulation of the methionine–homocysteine cycle and differences in terms of the energetic signature, with sickness impinging on glycolysis intermediates whilst rebound increased the triphosphorylated form of nucleotides. These findings indicate that rebound following sleep deprivation stimulates an energy rich setting in the brain that is devoid during sickness sleep.

## Graphical abstract

Different physiological sleep conditions impact the cortical metabolome. Two hours after sleep deprivation or intraperitoneal LPS injection, mice go to sleep. The cortical metabolome reflects changes during these physiological conditions. Specifically (a) levels of intermediates of the methionine–homocysteine cycle suggest a shift to hypomethylation during sickness and (b) elevation of ATP and GTP during rebound indicates energy replenishment.

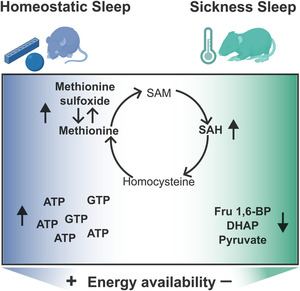

**Abbreviations**
AMP: adenosine monophosphate
ATP: adenosine triphosphate
COMT: catechol‐o‐methyltransferase
DHAP: dihidroxyacetone Phosphate
EEG: electroencephalogram
FDR: false discovery rate
GTP: guanosine triphosphate
GMP: guanosine monophosphate
HMDB: Human Metabolome Database
KEGG: Kyoto Encyclopedia of Genes and Genomes
LPS: lipopolysaccharide
NREM: non‐rapid eye movement
PPP: pentose phosphase pathway
PNMT: phenylethanolamine *N*‐methyltransferase
REM: rapid eye movement
SAH: S‐adenosylhomocysteine
SAM: S‐adenosylmethionine
SD: sleep deprivation

## 1 INTRODUCTION

Sleep is a conserved behavioural state observed in animals across the phylogenetic tree, but its manifestation is influenced by several factors. Based on prior wakefulness, age, sex and a variety of physiological contexts, such as pregnancy, chronic exercise or stress, sleep can increase or decrease and can even happen at times when wake would be more likely to occur (Cajochen et al., 2023; Duhart et al., 2023; Suchecki et al., 2012; Uchida et al., 2012). Still, there are differences such as which brain areas participate in each scenario and likely the purpose sleep is serving. In Drosophila, the requirement of sleep for appetitive memory depends upon food availability and uses different circuits under fed versus starved conditions (Chouhan et al., 2021), fear memory in mammals activates the amygdala to cause changes specifically in REM sleep (Wellman et al., 2016), and during sickness, a population of gabaergic cells in the lateral hypothalamus decreases their activity concomitantly with reduction of REM sleep (Borniger & de Lecea, 2021).

The most common model to study sickness consists of an inoculation of an inflammatory agent, the bacterial wall component lipopolysaccharide (LPS) (Engler et al., 2023; Krueger et al., 1986; Schedlowski et al., 2014). After a systemic challenge with LPS, cytokines increase (Zhang et al., 2024) and prolonged sleep deprivation causes a cytokine‐storm‐like syndrome reminiscent of severe inflammatory processes (Sang et al., 2023). Despite the shared humoral signals, the two types of sleep are remarkably different. Furthermore, during sickness, there is an increase in sleep fragmentation, REM decreases and NREM increases with also a reduction in delta power (Borniger & de Lecea, 2021; Ingiosi & Opp, 2016; Krueger et al., 1986; Trachsel et al., 1994), whilst during rebound following SD, both NREM and REM increase (Franken & Dijk, 2024; Noya et al., 2019).

One of the arguments for the benefits of sickness sleep behaviour is that energy is redirected for healing and immune support as opposed to waking activity (Ganeshan et al., 2019; Lochmiller & Deerenberg, 2000; Sadd & Schmid‐Hempel, 2009; Schmidt, 2014). Because the brain is a highly energy consuming organ that integrates information from the entire body (Peters et al., 2004), one could expect metabolic changes reflecting the reallocation of energy to support the immune activation.

To search for metabolic differences between normal sleep, rebound following SD and sickness sleep, we performed untargeted metabolomics on the cortex and hippocampus of mice during those three conditions and found that the cortex undergoes larger changes. Sleep deprivation and sickness had an impact on 19% of the brain metabolites identified. The methionine–homocysteine cycle was found to be regulated in different directions after SD and LPS, with methionine elevated during recovery sleep and high S‐adenosylmethionine levels after LPS challenge. Adenosine triphosphate (ATP) and other triphosphosphorylated metabolites were elevated during sleep after deprivation, suggestive of a replenishing scenario. By contrast, glycolysis intermediates were depleted during LPS, more indicative of a depleted condition. These results highlight the value of evaluating the cortical metabolic state under different sleep situations and get specific on the purpose of sleep.

## 2 MATERIALS AND METHODS

### 2.1 Animals

C57BL/6J male mice were acquired from Jackson Laboratory animal facility. Mice were given access to food and water ad libitum and were maintained under a 12‐h light–dark cycle. All mice were maintained in filter‐topped cages and given autoclaved food and water at the University of Pennsylvania University Laboratory Animal Resources (Penn ULAR) facility. All experiments were performed in accordance with the guidelines of the respective facilities and were approved by the regulations of the local institutional animal care and use committee (IACUC). At 8–10 weeks, animals were either challenged with an intraperitoneal injection of LPS (0.5 mg kg^−1^, O111:B4 Cayman 28872) or subjected to sleep deprivation (SD) for 4 h by gentle handling (when mice started to build nests or got quiet they were disturbed by introducing novel objects or introducing nesting material from another mouse). Two hours after LPS administration or the end of SD, mice were euthanized by cervical dislocation and cortex and hippocampus were isolated and flash frozen until processed.

### 2.2 Sample preparation

Frozen samples were weighted (10 mg) and homogenized using a motor pestle using an 0.45‐mL cold extraction solution (80% MeOH, 20% water with heavy‐labelled internal standard mix). Samples were centrifuged at 10,000*g* for 15 min at 4°C. Supernatant was collected and placed in dry ice for 30 min. Additional 200ul of extraction solution was added to pellet, centrifuged again and supernatant collected and combined with the previous batch. The combined samples were centrifuged again, transferred to cryovials and stored at −80°C.

### 2.3 LC‐MS/MS metabolomics

LC‐MS/MS was performed by the Proteomics and Metabolomics Core in The Wistar Institute, Philadelphia, PA. Samples were spiked with additional heavy‐labelled internal standards before analysis. One quality control (QC) pool sample was made by pooling a small aliquot from each sample. The QC pool was analysed at the beginning of the analysis and after every eight samples. Samples were analysed by LC‐MS/MS on a Q Exactive HF‐X mass spectrometer coupled to a ThermoScientific Vanquish LC System. Samples were analysed in a pseudorandomized order, and 4 μl of each sample or the QC pool was injected per run. Data were acquired with positive and negative polarity switching on the mass spectrometer. LC separation was performed under HILIC pH 9 condition using a ZIC‐pHILIC column.

### 2.4 Metabolomics data compound identification

Peak areas, representing metabolite levels, were extracted using ThermoScientific Compound Discoverer 3.3SP1. Metabolites were identified and quantified based on [M + H] + 1 and [M − H] − 1 adducts only. Metabolites were identified from a provided mass list and by MS/MS fragmentation of each metabolite followed by searching the mzCloud database (www.mzcloud.org). mzCloud matches that do not have corresponding mass list matches were required to have a minimum score of 50 in either the Reference (curated) or auto processed (noncurated) databases for Endogenous Metabolites and Steroids/Vitamins/Hormones compound classes. Pure compounds are required to confirm these compounds. For compounds identified in both positive and negative polarities, a single polarity was selected based on peak area and CV. Metabolites present near background levels based on extraction blanks are considered as background (sample peak area/control extraction peak area < 5). Quantitation was based on the equivalent percentage of material analysed for each sample. Values were then normalized to QC pool sample runs (QC Norm.) and subsequently by total signal of identified + filtered metabolites (QCSum Norm.).

### 2.5 Metabolomics data quality control

Coefficient of variation (CV) was calculated for the QC pool and for each experimental condition. Most metabolites in the QC pool and heavy‐labelled internal standards in the sample groups showed reproducible quantitation with CV ≤ 20%.

### 2.6 Multivariate data analysis

Analysis was performed using Simca‐P 17.0 software (Sartorius AG, Germany). Datasets were imported into the software, followed by unit variance scaling and mean centring. Principal component analysis (PCA) model was initially built to check for any obvious outlier and/or trend in the data and general data quality check. No noticeable outlier samples were observed. OPLS regression was carried out separately for cortex and hippocampus datasets using conditions (BL, SD and LPS) as the dependent variables and the metabolomic dataset as the independent variable. OPLS model was constructed using sevenfold cross‐validation. The robustness of the model was judged using Q2(cum) and CV‐ANOVA p statistic.

### 2.7 Pathway analysis

Significant metabolites from univariate and multivariate tests were used in metabolic pathway analyses through MetaboAnalyst 5.0 [44]. Metabolites were uploaded using HMDB identifiers and processed using a hypergeometric enrichment method, relative betweenness centrality for topology analysis using the *Mus musculus* (KEGG) pathway library. Significant pathways were defined using a FDR less than 0.2 (with those less 0.5 identified as potentially impacted pathways). To be included in analyses, an impact value greater than zero was required for at least one‐time comparison.

### 2.8 Sleep monitoring system

Sleep was monitored using a non‐invasive piezoelectric system (Signal Solution, Lexington, KY, USA). This method has been validated with electroencephalogram (EEG) and human scoring probing an accuracy of >90% discrimination of sleep and wake (Mang et al., 2014). The system consists on a cage with an open bottom that allows a direct contact of the animal with a piezoelectric sensor on the floor of the cage. The sensors allow the generation of a pressure signal. Output signals were amplified and filtered between 0.5 and 10 Hz. The amplified signals were analogue‐to‐digital (A/D) converted at a sampling rate of 128 Hz using the LabView 7.1 software (National Instruments, Austin, TX, USA). Piezo signals were analysed over tapered 8‐s windows at a 2‐s increment and a decision statistic was computed and classified using a linear discriminate classifier. The statistics reflects the regularity of the signal. The more regular the signal with peak spectral energy in the range typical of breathing (1 to 4 Hz) and lower relative amplitude, the more likely the mouse is in a sleep state (higher magnitude for the decision statistic). Data were binned at each hour on a rolling average percentage sleep. For sleep fragmentation, data were binned by length of individual bouts to calculate hourly mean bout length (in seconds).

## 3 RESULTS

### 3.1 Metabolic changes in the cortex reflect differences of unique sleep conditions

**FIGURE 1:**
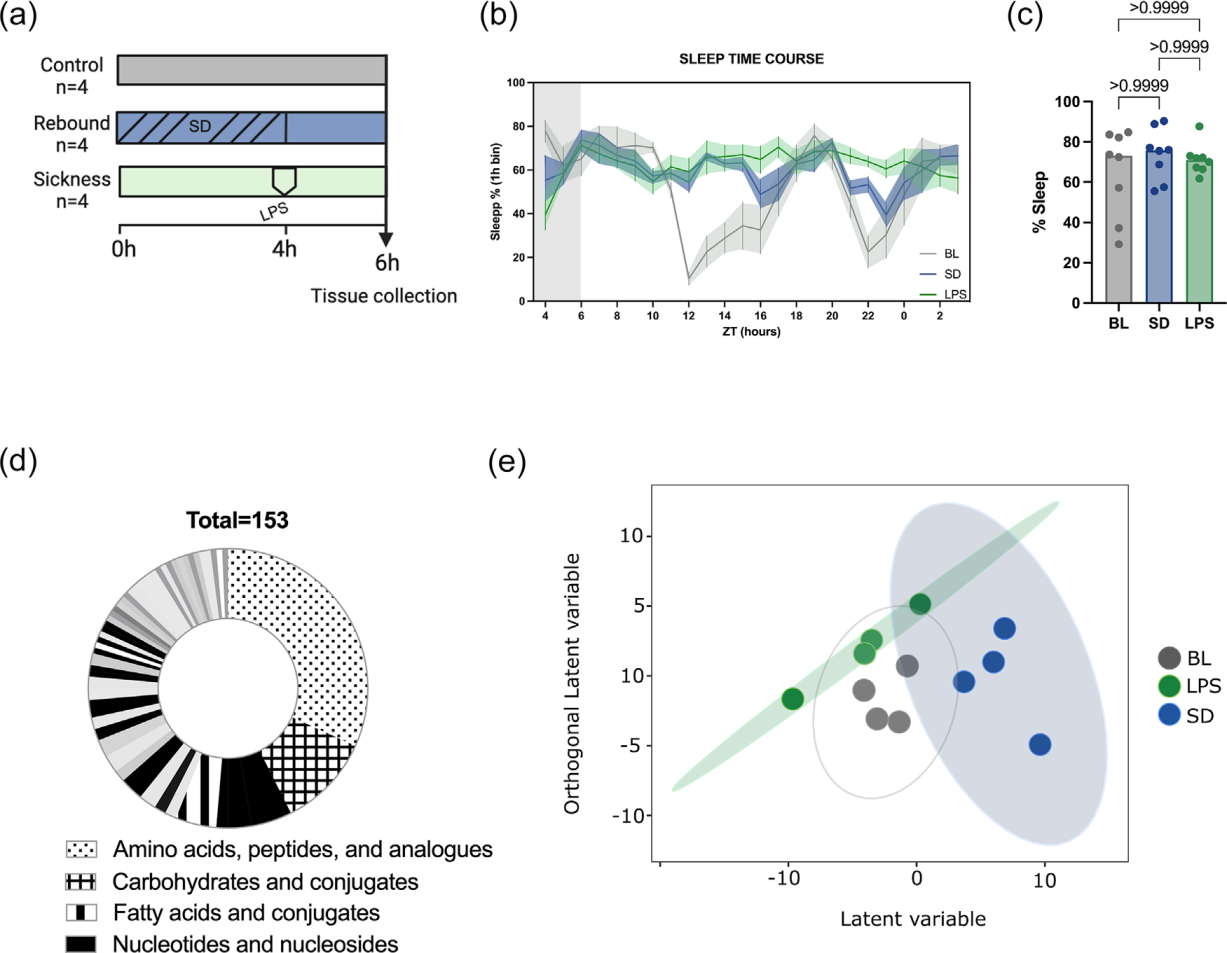
The cortical metabolome is modified by different physiological sleep types. (a) Workflow of the experimental design. Mice were divided into three conditions. Normal sleep or base line sleep (BL) consisted of animals that were allowed to sleep normally during the rest phase until sample collection at ZT6. Sleep rebound (SD) consisted of animals that were sleep deprived for 4 h from ZT0 to ZT4 and then allowed them to sleep two extra hours. Sickness sleep (LPS) included animals that were allowed to sleep from ZT0 until ZT4, when they were injected intraperitoneally with LPS and then allowed to sleep for 2 h. Cortex and hippocampus were collected at ZT6 including four mice per group. Samples were processed and analysed for untargeted metabolomics, analysed and preprocessed. (b) Sleep time course of the three conditions included in the study. The end of the grey area corresponds to the time when samples were harvested (*n* = 8). (c) Sleep percentage corresponding to the binned hour at the time of sample collection (*n* = 8, Kruskal–Wallis followed by Dunn's multiple comparison). (d) Overrepresentation analysis of metabolite sets in the filtered dataset. (e) OPLSDA of the cortex across the three sleep groups. Each point is a biological sample and the position of each point represents the multivariate concentration of the total filtered metabolites. Closer positions indicate similar metabolite levels. The model was evaluated by CV‐ANOVA.

To find physiological markers of sleep during sickness and rebound, we performed untargeted metabolomics on mouse cortex and hippocampus under three different conditions: baseline or normal sleep (BL), rebound following gentle deprivation (SD) and sleep post LPS challenge (LPS). The rebound condition consisted of 4 h of gentle sleep deprivation from ZT0 to ZT4 followed by 2 h of sleep. The sickness model was performed in parallel with an intraperitoneal injection of LPS (0.5 mg kg^−1^) at ZT4 and then 2 h of recovery (Figure 1a). With this design, we were able to analyse samples and capture the metabolic profile at a specific time of day (ZT6) eliminating the confound of circadian regulation of metabolism (Brown, 2016; Malik et al., 2020). Using a noninvasive monitoring system that uses a piezoelectric sensor to capture vibrations, we measured sleep in a parallel cohort of mice. We observed that after SD or LPS, mice progressively increased sleep to match that of control mice after 2 h (Figure 1b,c). The computed percentage of sleep in the hour around sample collection showed no difference in total sleep nor REM and NREM across the three samples (Figure S1‐1A,B). However, as expected from previous literature, we did see a reduction in REM in the LPS model in subsequent hours as well as increased fragmentation (Figure S1‐1C,D,E).

A total of 7472 compounds were detected of which 153 were annotated and quantified from both tissues (Table S1‐1). Amino acids, carbohydrates and nucleotides were the most abundant compounds and significantly enriched classes (Figure 1d, Table S1‐2).

Principal component analysis clearly differentiated the hippocampus and cortical samples (Figure S1‐2) indicating brain region specificity of metabolic profiles. This is consistent with other brain metabolomic studies where multiple regions display their own set of metabolites (Jiang et al., 2023; Vasilopoulou et al., 2016). Orthogonal partial least squares discriminant analysis (OPLS‐DA) was used to visualize the discriminate the three conditions in the cortex and hippocampus separately. The orthogonal variation to the variable class (BL, SD and LPS) was of higher magnitude for the cortex than the hippocampus indicating that sickness sleep, rebound and normal sleep have a larger effect on the cortical metabolome (Figure 1c). Given this observation we followed up on the cortex metabolome.

### 3.2 Shared metabolic trends arise during homeostatic rebound and sickness sleep

**FIGURE 2:**
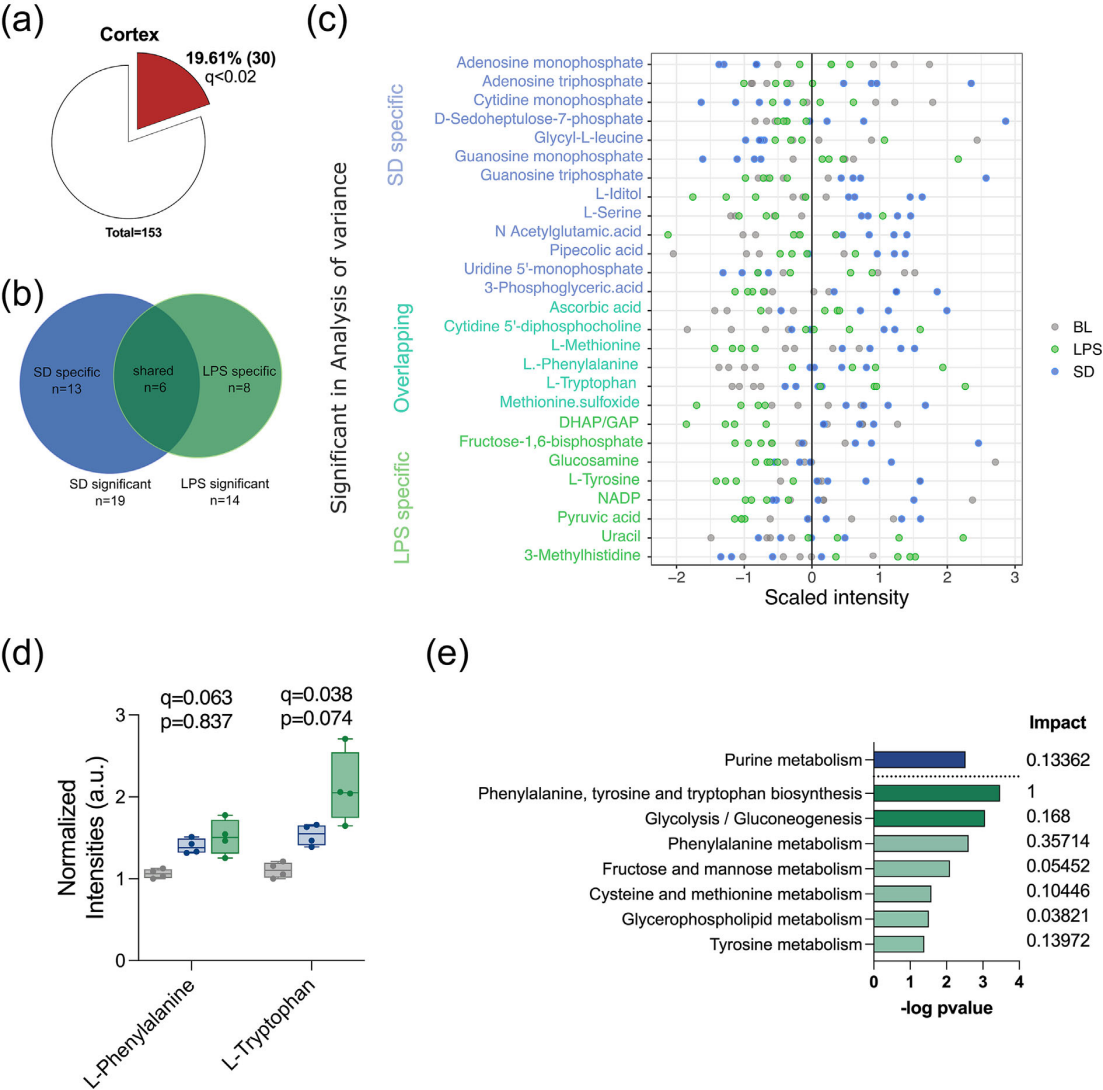
LPS and SD modulate specific sets of metabolites in the cortex. (a) Pie chart representing the significant metabolites in the cortex evaluated by CV‐ANOVA. (b) Venn diagram showing the overlap of significant features in the cortex that were specific for either SD or LP when compared with BL. (c) Scaled intensity values for the 30 significant metabolites according to the CV‐ANOVA. (d) Box plot of the normalized intensities for L‐phenylalanine and L‐tryptophan (*q* value corresponds to the CV‐ANOVA, *p* value indicates the pairwise comparison between SD and LPS). (e) Significant pathways enriched in the SD (blue) and LPS (green) metabolite sets including the common features. Pathways with an fdr <0.2 shown in darker colour and fdr <0.5 in light colour.

To discover the specific features that participate in the segregation of the three sleep conditions in the cortex we looked at metabolites that differentiated sickness sleep (LPS) and deprivation induced rebound (SD) from normal sleep (BL). Analysis of variance retrieved 30 compounds with significant differences between groups (Figure 2a, Table S2‐1); 19 and 14 metabolites differentiated the SD and LPS samples from BL, respectively, and six were differentially present in both (Figure 2b). Although the hippocampus metabolome was largely unchanged, there were three metabolites that reached significance in the CV‐ANOVA (Figure S2‐1A, Table S2‐2). These three metabolites were also significant in the cortex and they shared the same trend in both tissues (Figure S2‐1B).

Sleep deprivation promoted increases and decreases of specific metabolites whilst almost all LPS specific metabolites tended to be reduced (Figure 2c, Table S2‐1).

To explore the commonalities of SD and LPS induced sleep respect to BL, we looked at the six overlapping metabolites in the SD and LPS group that also had the same directionality of change. Four of these showed higher intensities in both the SD and LPS groups when compared with BL (Figure S2‐2A). L‐phenylalanine and L‐tryptophan showed the largest differences of the four (Figure 2d; *q* = 0.038 and *q* = 063). In addition, pathway analysis indicated that the phenylalanine, tyrosine and tryptophan biosynthesis pathway was the most significant category altered during sickness sleep but not during rebound (Figure 2e, Table S2‐3, Table S2‐4). After LPS, L‐tyrosine, a precursor of phenylalanine and tryptophan, was significantly reduced (Figure S2‐2B). All these findings indicate that tryptophan metabolism is altered during recovery sleep and sickness and likely more relevant for the latter.

### 3.3 The homocysteine–methionine cycle is differentially modulated during rebound and sickness sleep

**FIGURE 3:**
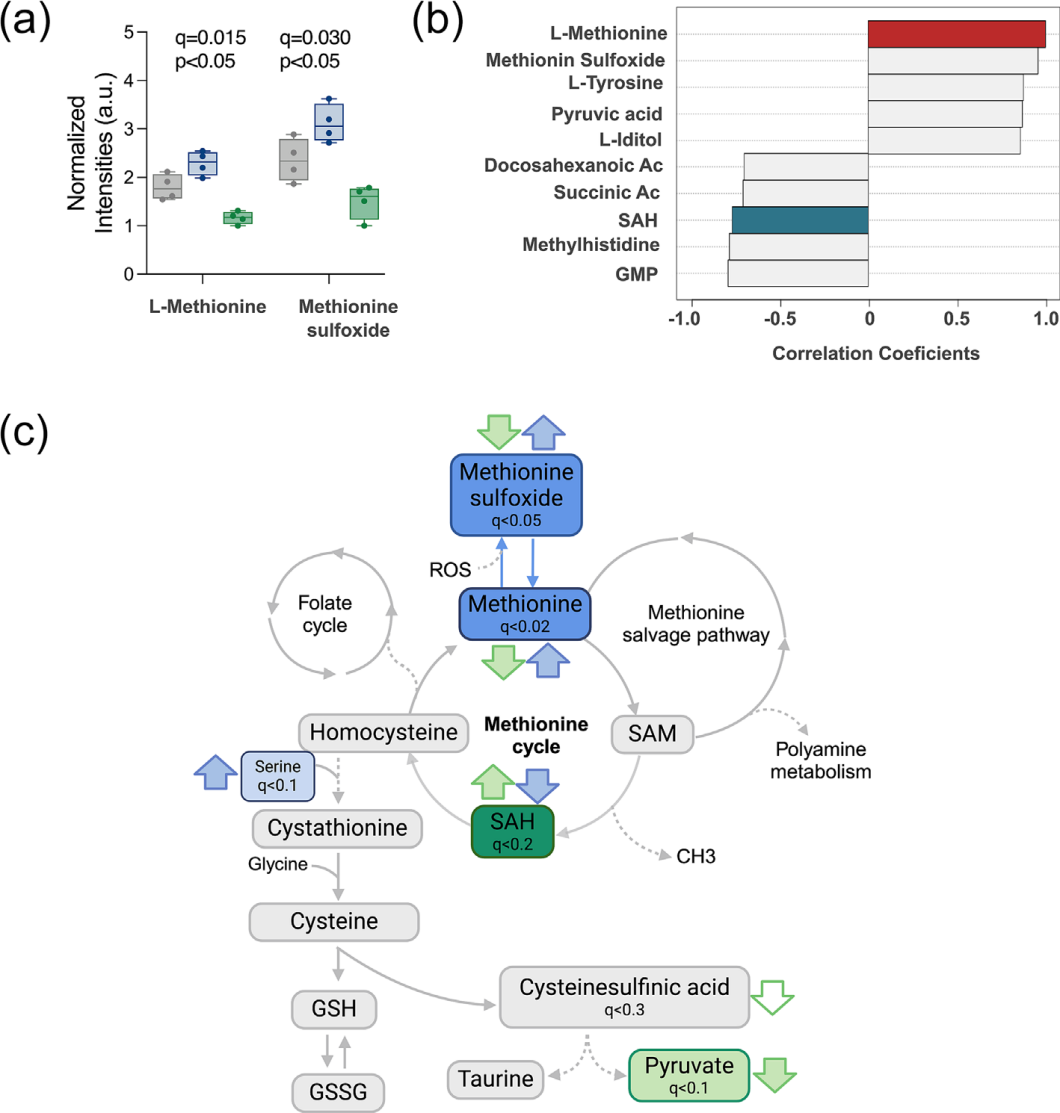
The methionine and homocysteine cycle is differentially modulated in during rebound and sickness sleep. (a) Box plot of the normalized intensities for L‐methionine and methionine sulfoxide (*q* value corresponds to the CV‐ANOVA, *p* value indicates the pairwise comparison between SD and LPS). (b) Result of the pattern analysis representing the top 10 peaks correlated with methionine including the positively correlated in red and the negatively correlated in blue. (c) Diagram of the methionine–homocysteine cycle with the main related pathways and outputs. Coloured boxes indicate significant metabolites found in the dataset. Arrows indicate the levels of the metabolite respect to BL, in green for LPS and in blue for SD. Filled arrows correspond to changes with an fdr <0.2 and empty arrows to an fdr <0.5.

Methionine and methionine sulfoxide increased after sleep deprivation and decreased during sickness when compared with normal sleep (Figure 3c). These two metabolites ranked at the top of those most significantly changed (Figure S2‐2A). To identify pathways that could be linked to this observation, we examined metabolites that had an anticorrelation profile to methionine. We found changes in SAH (Figure 3b), a potent methyltransferase inhibitor. Methionine and homocysteine metabolites are the core of the homocysteine–methionine cycle, the main provider of methyl groups in the cell crucial to many cellular functions, including polyamine synthesis, DNA synthesis, redox balance, and DNA and histone methylation (Shen et al., 2020). During rebound sleep, SAH is reduced and methionine increased, as is methionine sulfoxide, its oxidized form. Accumulation of methionine sulfoxide is a cellular strategy to protect from oxidative stress (Bender et al., 2008; Lourenço dos Santos et al., 2018), which increases during sleep deprivation and wakefulness and is released during sleep (Haynes et al., 2024; Noya et al., 2019).

In our sickness model (LPS), the reduction in methionine metabolites and the elevated levels of SAH indicate a drift in the homocysteine–methionine cycle. Accumulation of SAH leads to the synthesis of cysteine and ultimately glutathione (GSH) to regulate redox homeostasis (Lee & Gladyshev, 2011). Cysteine was not detected in our dataset; however, other metabolites related to the cycle were, although many did not show differences between groups.

Interestingly, ascorbic acid, a potent antioxidant, was also found increased in both the SD and LPS group (Figure S3‐1). All in all, this strongly indicates that sleep deprivation and sickness impose a differential regulation on the methionine–homocysteine cycle with both strategies potentially increasing the antioxidant potential of the brain (Figure 3c).

### 3.4 Opposite trends in core energy pathways during sickness and rebound

**FIGURE 4:**
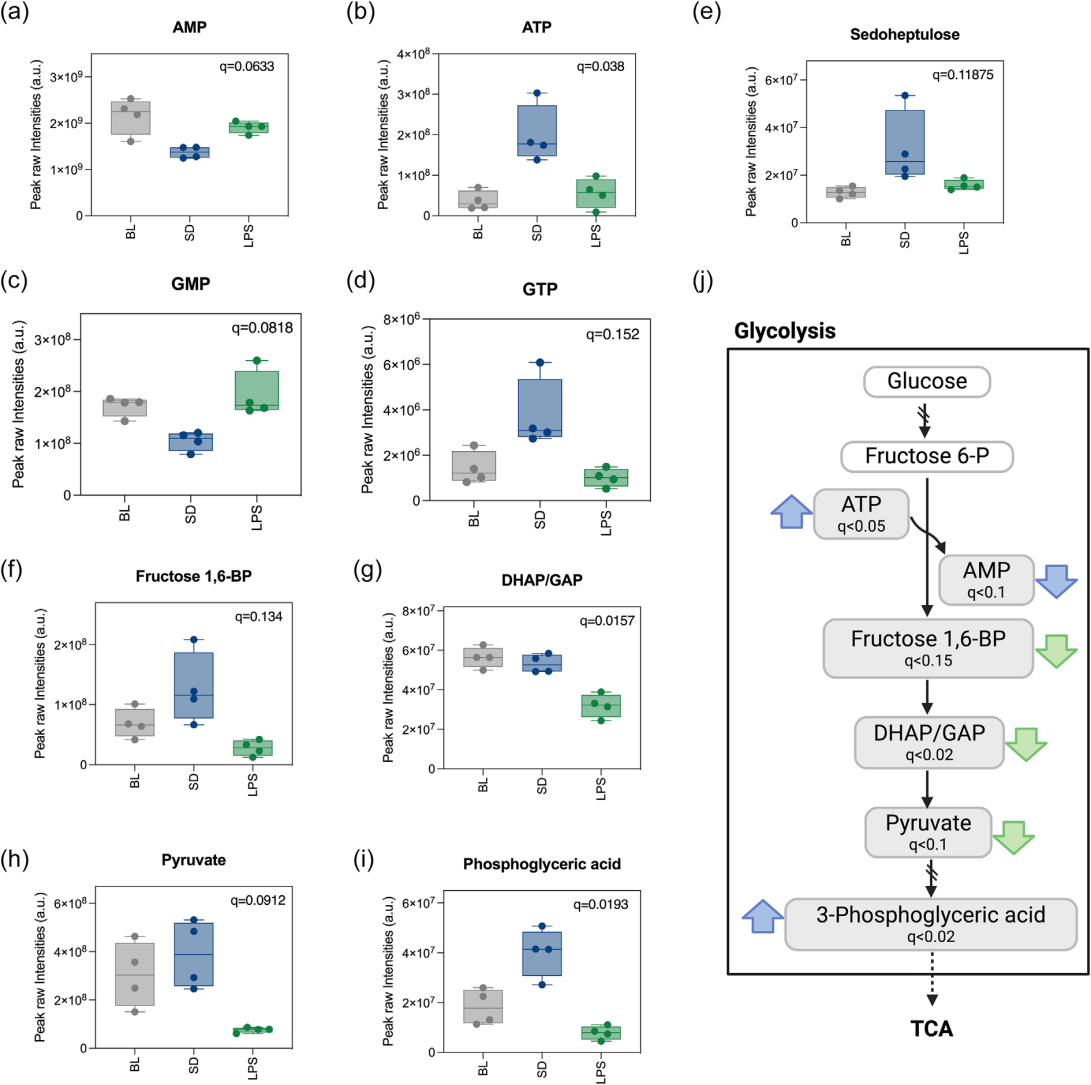
Metabolites related to energetic pathways differentiate rebound and sickness sleep. (a–i) Boxplot representing the intensity levels of significant metabolites related to energy metabolic pathways. Significance was evaluated with a CV‐ANOVA, and *q* values from the analysis are depicted. (j) Representation of the glycolytic pathway and PPP showing significant metabolites found in our dataset. Filled arrows indicate the directionality of significant change for LPS in green and SD in blue for metabolites with and frd <0.2. Empty arrows indicate the same for metabolites with an fdr <0.5.

Purine metabolism was the only significantly enriched pathway in the SD metabolite group (Figure 2e). Nucleotide, the metabolites included in the pathway, constituted half of the SD significant features (Figure 2c). The monophosphate forms of cytidine, uridine, guanosine and adenosine showed a negative fold change whilst the triphosphorylated forms of adenosine and guanosine a positive one (Figure 4a–d). In addition, the pentose phosphate pathway (PPP) is a main source of precursors for nucleotide biosynthesis and consistent with the increased nucleotide levels in SD, the PPP intermediate sedoheptulose‐7P was significantly increased in SD (Figure 4e). Thus, during rebound sleep, there appears to be a replenishment of resources in the brain in the form of energy exchange molecules.

Half of the LPS significant metabolites were components of glycolysis/gluconeogenesis and were reduced compared with the other two conditions. These included three compounds that participate in reactions downstream of the rate‐limiting step of glycolysis: dihidroxiacetone phosphate (DHAP), fructose 1,6‐biphosphate and pyruvate (Figure 4g–i). By contrast, 3‐phosphoglyceric acid, an intermediate belonging to the end of the glycolytic cascade, was high in SD (Figure 4j), suggesting opposing effects of homeostatic rebound and sickness sleep on the utilization of main energy pathways (Figure 4k).

## 4 DISCUSSION

Many studies have focused on comparing homeostatic sleep rebound to normal sleep. However, few have emphasized a side‐by‐side evaluation of the sleep that occurs under diverse physiological states. Indeed, how molecular underpinnings of sleep vary in these different situations is largely unknown. Here, we performed untargeted metabolomics on the cortex and hippocampus from mice during their normal rest phase, unperturbed or following sleep deprivation or challenge with the endotoxin LPS. What the results show is that with both manipulations, the cortical metabolic landscape is affected to a larger extent than the hippocampus. Some pathways are altered in the same direction whilst others show opposite effects. The methionine–homocysteine cycle stood out as a clear hallmark that distinguished rebound and sickness sleep, supporting the idea of a hypomethylation environment in the latter. More broadly, the data indicate rebound to sleep deprivation as an energetically rich framework with a high abundance of triphosphorylated nucleotides as opposed to an energy depleted scenario during sickness sleep.

Despite providing robust comparisons, steady‐state metabolomics measures end points and intermediates of metabolic processes and thus can mask some features such as the metabolic flux through different pathways. Thus, it does not necessarily reflect previous history but rather a real‐time image of the metabolic landscape (Malik et al., 2020). For instance, intermediates of the methionine–homocysteine cycle show opposite trends after LPS and SD (Figure 2). Methionine is reduced after LPS and increased after SD; by contrast, SAH is increased after LPS and reduced after SD. Although the nature of steady state metabolomics prevents us from ascertaining which part of the cycle is working at faster or slower rate, the data support the idea that multiple steps are affected. SAH is a potent methylation inhibitor and the increased levels after LPS suggest there might be a reduction in methyltransferase activities. Increased levels of SAH have been correlated with the severity of outcomes after a high LPS dose (Dai et al., 2016) and are also characteristic of Alzheimer's disease brains, potentially reducing the methyltransferase activity of catechol‐o‐methyltransferase (COMT) and phenylethanolamine *N*‐methyltransferase (PNMT), enzymes responsible for the catabolism and biosynthesis of catecholamines such as serotonin and dopamine (Kennedy et al., 2004; Ravaglia et al., 2005; Selley, 2007). Altered levels of serotonin and dopamine have been reported in different sickness models in peripheral tissues (de Bem Alves et al., 2021; Wong et al., 2023; Zager et al., 2018), and despite the fact that we did not detect these neurotransmitters with our methodology, it would be interesting to perform target metabolomics in our LPS model as well as evaluate the methyltransferase activity of COMPT and PNMT. Changes in SAH can also impact DNA methylation, and sleep has been shown to modulate DNA methylation in the brain, specifically in genes involved in synaptic regulation (Massart et al., 2014; Ventskovska et al., 2015). Based on our observations, it is likely that the cortical methylome is differently modulated in our experimental paradigms. It would be interesting to measure DNA methylation on synaptic genes and functional or structural implications.

In our experimental design, we aimed to compare samples from mice that were sleeping but in different paradigms. ZT6 is the midpoint of the normal rest phase of mice when most sleep pressure, measured as slow wave activity, is usually dissipated (Franken & Dijk, 2024). We used a noninvasive sleep monitoring system to measure sleep, and our data show that 2 h after gentle sleep deprivation or after LPS challenge, sleep increases to an equivalent level as that in the control group (Figure 1). However, the inflammation model is a complex model that induces increased sleep, increased fragmentation and suppression of REM as well as possible body temperature changes (Ingiosi & Opp, 2016). With our experiments, we cannot rule out the contribution of body temperature, but dissociating effects of sleep and temperature is challenging and will be the purpose of follow‐up studies.

One of the main conclusions from this project is how differently sickness and sleep deprivation affect the energetic framework of the sleep that follows. After sleep deprivation, we found higher levels of ATP and guanosine triphosphate (GTP) but lower levels of adenosine monophosphate (AMP) and guanosine monophosphate (GMP). This is consistent with previous findings regarding a surge in ATP levels in the initial hours of sleep (Dworak et al., 2010; Wong‐Riley, 2011). ATP levels have been positively correlated with increases in NREM and negatively correlated with increases in REM, which, together with other observations (Braun et al., 1997; Nofzinger et al., 2002), indicate a negative energy balance in the cortex during REM, likely due to high energy expenditure. During sickness sleep, REM is completely absent (Borniger & de Lecea, 2021; Morrow & Opp, 2005), raising the question of whether this phenomenon constitutes a strategy to preserve energy during sickness. If this is the case, sickness sleep may facilitate recovery from illness, but may not provide all the restorative benefits of normal/homeostatic sleep.

The idea of metabolites correlating with sleep and wake and even individual sleep states is not new. A study led by Professor Malcom Kohler and Steven Brown found that breath metabolites change rapidly between REM, NREM and wake (Nowak et al., 2021) with over 50% of the features detected being regulated by sleep stage. In that study, during REM sleep, intermediates of the TCA cycle and pyruvate increased, likely due to an activation of glycolysis. In our LPS model, we found decreased glycolysis intermediates as well as pyruvate which would be in line with a reduction in REM. With our sleep monitoring system, we did not find a decrease in REM at the time of sample collection; however, it occurred in the subsequent hours. The fact that we don't see a suppression of REM shortly after, could be due to a lack of sensitivity (Borniger & de Lecea, 2021; Morrow & Opp, 2005). It would be interesting to investigate why the energetic state associated with REM is different.

The purpose of sleep remains a question of intense interest that seems to have multiple answers. This may be reflected in the fact that sleep manifests in very different contexts, each of which may have unique requirements of sleep. Therefore, evaluating these divergent sleep situations may provide insight into the various functions of sleep. In this study, we aimed to compare three different sleep paradigms; normal sleep, rebound following sleep deprivation and sickness sleep. We performed untargeted metabolomics in cortical samples from mice and found a differential modulation in 19% of the identified metabolites. Our findings underscore the need to dissect the cellular and molecular basis of sleep under different conditions in order to gain a comprehensive account of sleep function.

### 4.1 Limitations of the study

Some study limitations should be acknowledged. The small sample size might have compromised the depth of the findings by impacting the statistical power. For future experiments of this type, we would recommend increasing the sample size to an *n* = 6–8. Notwithstanding this weakness, this study has shown differences in the cortical metabolome of mice that are experiencing rebound sleep or sleep during sickness, relative to normal daily sleep, in the same direction or in the opposite. Additional conditions in the study design could have helped to account for some variables introduced by LPS challenge. For instance, because this challenge not only increases sleep but also induces changes in body temperature, a group of mice that were kept awake for 2 h after LPS would be valuable. In addition, including three groups at ZT4 (end of SD and time of injection) would have helped to elucidate the dynamics of metabolic changes.

## AUTHOR CONTRIBUTIONS

Amita Sehgal and Sara B. Noya designed the research; Zhifeng Yue and Sara B. Noya conducted the experiments; Sara B. Noya and Arjun Sengupta analysed data; Amita Sehgal and Sara B. Noya wrote the first draft of the paper; Amita Sehgal supervised the study. Amita Sehgal, Aalim Weljie, Arjun Sengupta, and Sara B. Noya reviewed and edited the paper.

## CONFLICT OF INTEREST STATEMENT

The authors declare no competing financial interests.

### PEER REVIEW

The peer review history for this article is available at https://www.webofscience.com/api/gateway/wos/peer-review/10.1111/ejn.16588.

## Supporting information


**Figure S1‐1.** **A and B**. NREM and REM percentage corresponding to the binned hour at the time of sample collection (n = 8). **C and D**. NREM and REM course of the three conditions included in the study. The end of the grey area corresponds to the time when samples were harvested. **D**. Median sleep bout duration from ZT4 until ZT6. (n = 8, Kruskal‐Wallis followed by Dunn's multiple comparison).
**Figure 1–2.** PCA of all the cortex and hippocampus samples. Each point is a biological sample and the position of each point represents the multivariate concentration of the total filtered metabolites. Closer positions indicate similar metabolite levels. PC2 explains the difference between cortex and hippocampus. PC2 explains the difference between the sleep conditions.

**Figure S2‐1.** **A**. Pie chart representing the significant metabolites in the hippocampus evaluated by CV‐ANOVA, q < 0.2. **B**. Boxplot representing the intensity levels of common significant metabolite in the cortex and hippocampus (CV‐ANOVA, q < 0.2). **C**. Box plot of the normalized intensities for the 6 features that were differentially regulated in both SD and LPS when compared to BL (q value corresponds to the CV‐ANOVA, p‐value indicates the pairwise comparison between SD and LPS).

**Table S1‐1.** 153 mass list matches. Background compounds, compounds poorly detected in QCs, potential in‐source fragments, potential duplicate entries, and heavy‐labeled internal standards were removed from these results. Values are normalized values (to QC pool sample runs and subsequently to total signal of identified+filtered metabolites).

**Table S1‐2.** Metabolic enrichment from 153 metabolites using the Enrichment Over Representation Analysis option in Metaboanalyst.

**Table S2‐1.** CV‐ANOVA for Cortex. In red metabolites with fdr < 0.2. In blue metabolites with fdr < 0.3.

**Table S2‐2.** CV‐ANOVA for Hippocampus. In red metabolites with fdr < 0.2.

**Table S2‐3.** Pathway analysis for SD significant metabolites in the cortex performed using Metaboanalyst with the mouse KEGG library. In red significant pathways.

**Table S2‐4.** Pathway analysis for LPS significant metabolites in the cortex performed using Metaboanalyst with the mouse KEGG library. In red significant pathways.

**Table S3‐1.** Result of pattern analysis using Methionine as a bait. The analysis was performed using Metaboanalyst.

## Data Availability

All data will be available through Metabolomics Workbench and on request.
