# Supplementary material for: Balancing brain metabolic states during sickness and recovery sleep
Source: Eur J Neurosci. 2024 Nov 14;60(11):6605–16. doi: 10.1111/ejn.16588 (PMC11612838; doi:10.1111/ejn.16588)

Figure 1-1. A and B. NREM and REM percentage corresponding to the binned hour at the time of sample collection (n=8). **C and D**. NREM and REM course of the three conditions included in the study. The end of the grey area corresponds to the time when samples were harvested. **D**. Median sleep bout duration from ZT4 until ZT6. (n=8, Kruskal-Wallis followed by Dunn's multiple comparison).

Figure 1-2. PCA of all the cortex and hippocampus samples. Each point is a biological sample and the position of each point represents the multivariate concentration of the total filtered metabolites. Closer positions indicate similar metabolite levels. PC2 explains the difference between cortex and hippocampus. PC2 explains the difference between the sleep conditions.

Figure 2-1. A. Pie chart representing the significant metabolites in the hippocampus evaluated by CV-ANOVA, q<0.2. **B**. Boxplot representing the intensity levels of common significant metabolite in the cortex and hippocampus (CV-ANOVA, q<0.2). **C**. Box plot of the normalized intensities for the 6 features that were differentially regulated in both SD and LPS when compared to BL (q value corresponds to the CV-ANOVA, p-value indicates the pairwise comparison between SD and LPS).

Table 1-1. 153 mass list matches. Background compounds, compounds poorly detected in QCs, potential in-source fragments, potential duplicate entries, and heavy-labeled internal standards were removed from these results. Values are normalized values (to QC pool sample runs and subsequently to total signal of identified+filtered metabolites).

Table 1-2. Metabolic enrichment from 153 metabolites using the Enrichment Over Representation Analysis option in Metaboanalyst.

Table 2-1. CV-ANOVA for Cortex. In red metabolites with fdr<0.2. In blue metabolites with fdr<0.3.

Table 2-2. CV-ANOVA for Hippocampus. In red metabolites with fdr<0.2.

Table 2-3. Pathway analysis for SD significant metabolites in the cortex performed using Metaboanalyst with the mouse KEGG library. In red significant pathways.

Table 2-4. Pathway analysis for LPS significant metabolites in the cortex performed using Metaboanalyst with the mouse KEGG library. In red significant pathways.

Table 3-1. Result of pattern analysis using Methionine as a bait. The analysis was performed using Metaboanalyst.

Figure 1-1

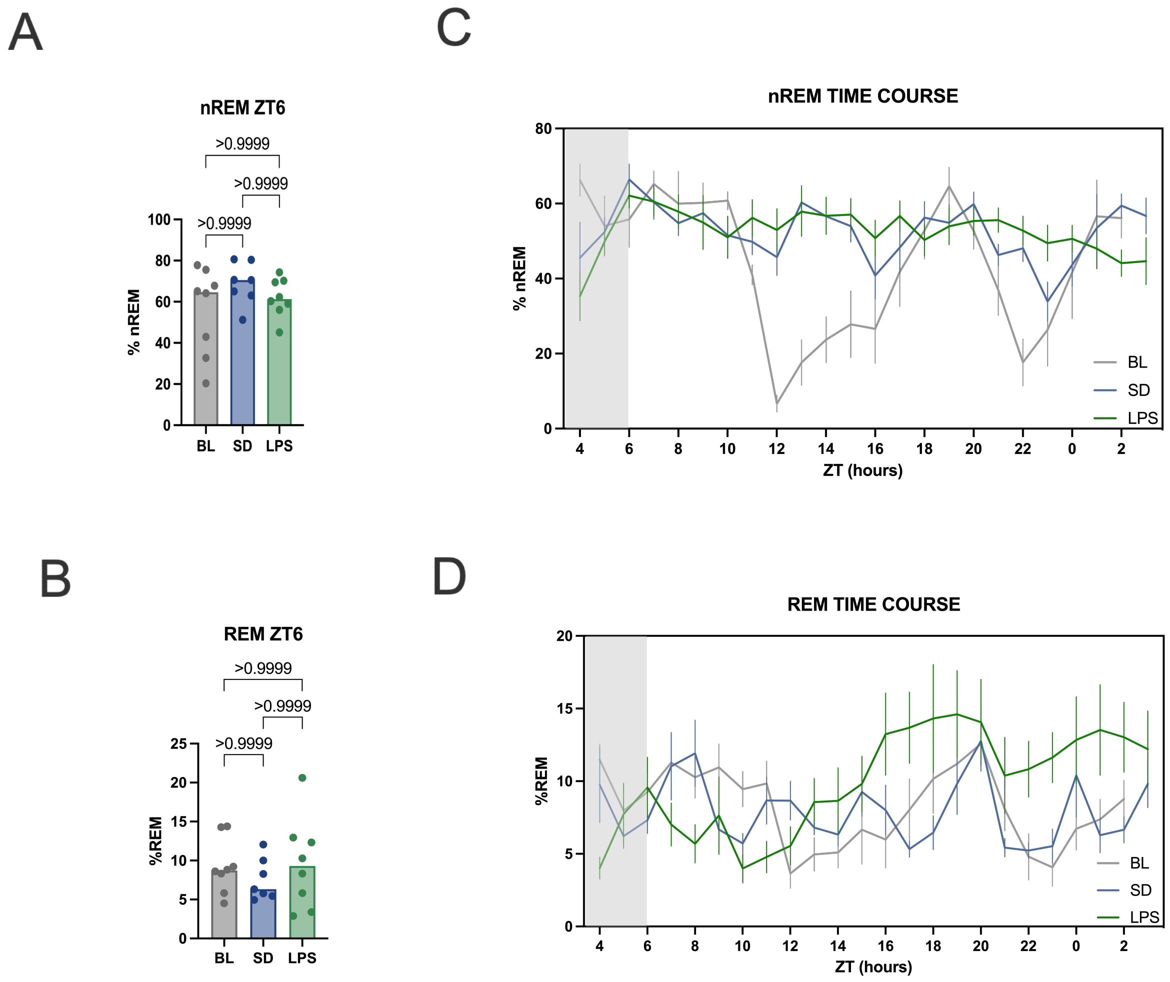
A
nREM ZT6
>0.9999
>0.9999
>0.9999
% nREM
100
80
60
40
20
0
BL
SD
LPS
B
REM ZT6
>0.9999
>0.9999
>0.9999
%REM
25
20
15
10
5
0
BL
SD
LPS
C
nREM TIME COURSE
% nREM
80
60
40
20
0
4
6
8
10
12
14
16
18
20
22
0
2
ZT (hours)
BL
SD
LPS
D
REM TIME COURSE
%REM
20
15
10
5
0
4
6
8
10
12
14
16
18
20
22
0
2
ZT (hours)
BL
SD
LPS

Figure 1-2

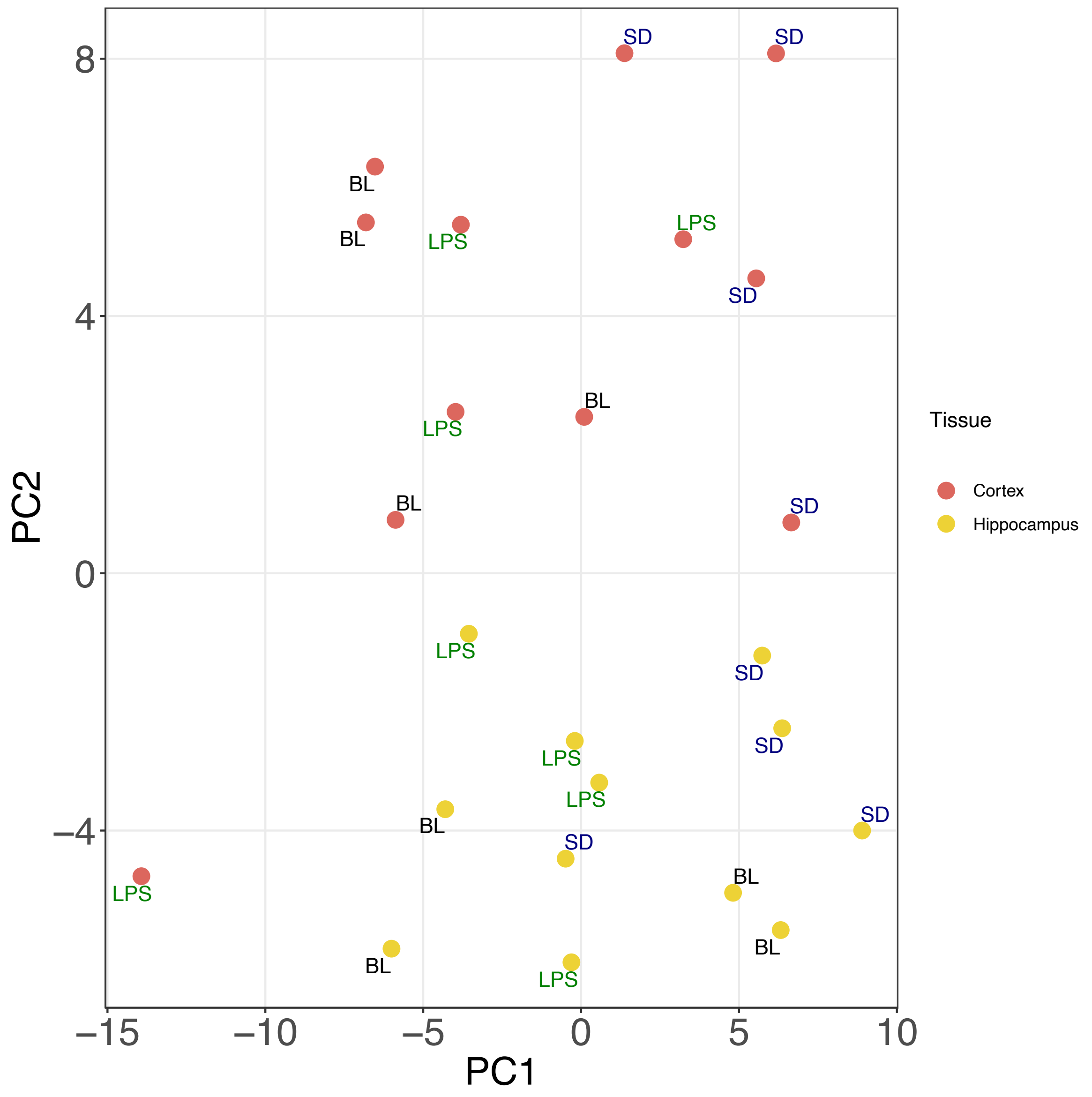

Supplement: Supplementary file 1 — Figure S1‐1. A and B. NREM and REM percentage corresponding to the binned hour at the time of sample collection (n = 8). C and D. NREM and REM course of the three conditions included in the study. The end of the grey area corresponds to the time when samples were harvested. D. Median sleep bout duration from ZT4 until ZT6. (n = 8, Kruskal‐Wallis followed by Dunn's multiple comparison). Figure 1–2. PCA of all the cortex and hippocampus samples. Each point is a biological sample and the position of each point represents the multivariate concentration of the total filtered metabolites. Closer positions indicate similar metabolite levels. PC2 explains the difference between cortex and hippocampus. PC2 explains the difference between the sleep conditions. [file EJN-60-6605-s007.pdf]
