# Supplementary figures and images for: Balancing brain metabolic states during sickness and recovery sleep
Source: Eur J Neurosci. 2024 Nov 14;60(11):6605–16. doi: 10.1111/ejn.16588 (PMC11612838; doi:10.1111/ejn.16588)

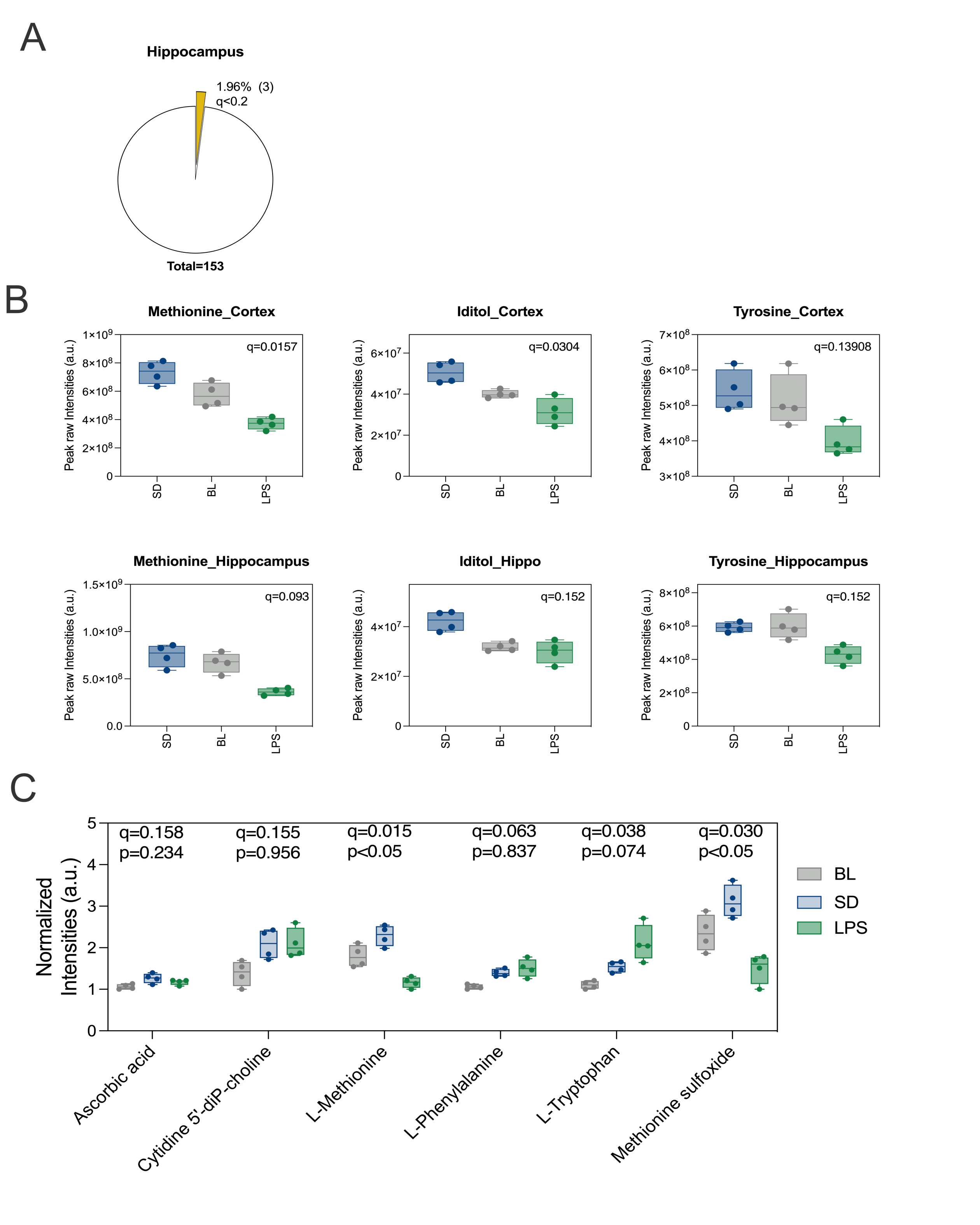

Supplement: Supplementary file 2 — Figure S2‐1. A. Pie chart representing the significant metabolites in the hippocampus evaluated by CV‐ANOVA, q < 0.2. B. Boxplot representing the intensity levels of common significant metabolite in the cortex and hippocampus (CV‐ANOVA, q < 0.2). C. Box plot of the normalized intensities for the 6 features that were differentially regulated in both SD and LPS when compared to BL (q value corresponds to the CV‐ANOVA, p‐value indicates the pairwise comparison between SD and LPS). [file EJN-60-6605-s004.tiff]
